# Cardioprotective Effects of PARP Inhibitors: A Re-Analysis of a Meta-Analysis and a Real-Word Data Analysis Using the FAERS Database

**DOI:** 10.3390/jcm13051218

**Published:** 2024-02-21

**Authors:** Ja-Young Han, Young-Eun Seo, Jae-Hee Kwon, Jae Hyun Kim, Myeong Gyu Kim

**Affiliations:** 1 Graduate School of Pharmaceutical Sciences, Ewha Womans University, Seoul 03760, Republic of Korea; 2 Graduate School of Clinical Biohealth, Ewha Womans University, Seoul 03760, Republic of Korea; yeunv@naver.com; 3 School of Pharmacy, Jeonbuk National University, Jeonju 54896, Republic of Korea; kimkimjh@jbnu.ac.kr; 4 College of Pharmacy, Ewha Womans University, Seoul 03760, Republic of Korea

**Keywords:** PARP inhibitors, cardiac adverse events, combination, meta-analysis, FAERS

## Abstract

**Objective:** This study aimed to assess the potential of PARP inhibitors to prevent cardiotoxicity. **Methods:** First, a re-analysis and update of a previously published study was conducted. Additional searches were conducted of the PubMed and Cochrane Central Register of Controlled Trials databases on 2 June 2023. After the selection process, the pooled odds ratio (OR) for cardiac adverse events (AEs) was calculated. Second, the FAERS database was examined for 10 frequently co-administered anticancer agents. The reporting odds ratio (ROR) was calculated based on the occurrence of cardiac AEs depending on the co-administration of PARP inhibitors. **Results:** Seven studies were selected for the meta-analysis. Although not statistically significant, co-administration of PARP inhibitors with chemotherapy/bevacizumab decreased the risk of cardiac AEs (Peto OR = 0.61; *p* = 0.36), while co-administration with antiandrogens increased the risk of cardiac AEs (Peto OR = 1.83; *p* = 0.18). A total of 19 cases of cardiac AEs were reported with co-administration of PARP inhibitors in the FAERS database. Co-administration of PARP inhibitors with chemotherapy/bevacizumab significantly decreased the risk of cardiac AEs (ROR = 0.352; 95% confidence interval (CI), 0.194–0.637). On the other hand, for antiandrogens co-administered with PARP inhibitors, the ROR was 3.496 (95% CI, 1.539–7.942). The ROR for immune checkpoint inhibitors co-administered with PARP inhibitors was 0.606 (95% CI, 0.151–2.432), indicating a non-significant effect on cardiac AEs. **Conclusion:** This study reports that PARP inhibitors show cardioprotective effects when used with conventional anticancer agents.

## 1. Introduction

Poly(adenosine diphosphate [ADP] ribose) polymerase (PARP) inhibitors have emerged as a promising class of therapeutic agents with remarkable efficacy in cancer treatment due to their ability to inhibit DNA repair through PARP. Inhibition of PARP leads to the accumulation of oxidative stress, single-strand breaks, and subsequent double-strand breaks [1]. This mechanism makes PARP inhibitors particularly effective in treating DNA repair-deficient cancers, including breast cancer with *BRCA* mutations [1].

In addition to their anticancer effects achieved through a different mechanism from conventional anticancer agents, PARP inhibitors also provide benefits in terms of mitigating adverse reactions caused by these agents. A large number of anticancer agents, including anthracyclines, taxanes, and alkylating agents, have been found to induce cardiotoxicity by causing oxidative stress and DNA damage in myocytes and endothelial cells [2]. As a result, cardiotoxicity can manifest in various forms, such as congestive heart failure and myocardial infarction. Interestingly, PARP is activated in response to sensing DNA damage in myocytes and endothelial cells, which leads to functional impairment and death of these cells [3]. Activation of PARP is an essential mechanism for maintaining genomic integrity. However, excessive and sustained activation of PARP can deplete nicotinamide adenine dinucleotide (NAD+) and adenosine triphosphate (ATP), resulting in cellular dysfunction and ultimately cell death in cardiac and endothelial tissues [3,4].

The use of PARP inhibitors can fortunately help counteract detrimental effects by preserving intracellular NAD+ and ATP, preventing cell death in the cardiovascular system. Furthermore, PARP inhibitors have demonstrated anti-inflammatory effects, further contributing to their potential cardioprotective benefits [3]. Considering these findings, combining PARP inhibitors with conventional anticancer agents holds promise for providing dual benefits: enhancing the effectiveness of cancer treatment and providing cardioprotection against the potential cardiotoxicity associated with some anticancer agents.

Recent animal studies have provided intriguing insights into the potential cardioprotective effects of PARP inhibitors that extend beyond their major anticancer effects. For instance, Pacher et al. demonstrated that PARP inhibitors significantly improved cardiac function and reduced mortality in a doxorubicin-induced heart failure model [5,6]. Similarly, Bartha et al. showed that PARP inhibitors attenuated doxorubicin-induced cardiac injury by modulating kinase pathways and heat shock protein expression in murine models [7]. In addition, Szenczi et al. showed that PARP inhibitors prevented modulation of myocardial contractile disturbances and calcium overload in doxorubicin-treated rats [8].

Despite these promising findings in animal studies, the current research on the cardioprotective effects of PARP inhibitors in humans is limited. While one study on INO-1001, a PARP inhibitor, has shown promising results with respect to improving reperfusion injury, there is still a lack of direct evidence regarding the cardioprotective effects of PARP inhibitors against cardiotoxicity caused by anticancer agents [9]. One meta-analysis has attempted to assess the relationship between major adverse cardiac events (MACEs) and PARP inhibitors using data from studies conducted up to 30 April 2022 [10]. The findings revealed that combining PARP inhibitors with chemotherapy did not significantly increase the risk of MACEs (*p* = 0.24). However, when PARP inhibitors were combined with antiandrogens, there was a notable increase in the risk of MACEs (Peto odds ratio [OR] = 1.71; *p* = 0.02) [10]. However, this meta-analysis primarily focused on evaluating the potential cardiovascular toxicity of PARP inhibitors. Further research is needed to specifically investigate the cardioprotective effects of PARP inhibitors when used in combination with various anticancer agents.

This study aimed to assess the potential of PARP inhibitors to prevent cardiotoxicity caused by other anticancer drugs. To achieve this, a re-analysis of this published meta-analysis was conducted, and data from the U.S. Food and Drug Administration Adverse Event Reporting System (FAERS) database were also analyzed.

## 2. Materials and Methods

### 2.1. Re-Analysis of a Previous Meta-Analysis

A re-analysis and update of a previously published meta-analysis was conducted [10]. Table S1 presents the population, intervention, comparison, outcome, and study design (PICOS) corresponding to the questions addressed in this study. In contrast to the previous meta-analysis, certain terms such as arrhythmia, tachycardia, and bradycardia were excluded from the outcomes, and additional MedDRA terms related to cardiotoxicity were incorporated.

Since the search date of the previous study (1 May 2022), additional searches were conducted of the PubMed and Cochrane Central Register of Controlled Trials on 2 June 2023, using the search terms listed in the Table S2. For inclusion in this meta-analysis, studies were required to meet the PICOS inclusion criteria (Table S1). Furthermore, studies that were already included in the previous meta-analysis were also evaluated based on these inclusion criteria. Conference abstracts were included if they provided raw data on cardiotoxicity. The selection process involved two researchers (Y.E.S. and M.G.K.) who conducted two stages of screening: an initial screening based on the titles and abstracts, followed by a full-text assessment.

The total number of patients and incidence of cardiac adverse events in both the intervention and control groups were extracted to calculate the pooled OR for cardiac adverse events. Peto’s method was used to calculate the pooled OR [11]. Furthermore, subgroup analyses were performed based on the type of anticancer agents (chemotherapy/bevacizumab or androgen receptor inhibitors) and the severity of adverse events (grade ≥ 3 according to the National Cancer Institute Common Terminology Criteria for Adverse Events). The meta-analysis was conducted using RevMan version 5 software (The Cochrane Collaboration, Copenhagen, Denmark).

### 2.2. Analysis of the FAERS Database

The FAERS database, established by the Food and Drug Administration (FDA), serves as a spontaneous reporting system to support post-marketing surveillance by collecting global reports on adverse events. For the analysis, all records from the FAERS database covering a 10-year period from 1 January 2013 to 31 December 2022 were utilized. This study obtained IRB exemption approval (ewha-202304-0013-01).

For this study, five PARP inhibitors (niraparib, olaparib, rucaparib, talazoparib, and veliparib) were selected for evaluation. To identify frequently co-administered anticancer agents with PARP inhibitors, the DRUG files in the FAERS database were examined, and the ingredient names of all drugs reportedly used in combination with these five PARP inhibitors were collected. Subsequently, 10 anticancer agents including bevacizumab, nivolumab, carboplatin, atezolizumab, paclitaxel, abiraterone acetate, durvalumab, doxorubicin, pembrolizumab, and temozolomide were chosen for the analysis. Metoprolol was also frequently co-administered with PARP inhibitors; however, it was excluded from the analysis as it is not an anticancer agent.

Cardiotoxicity was defined based on the preferred terms (PTs) associated with cardiac adverse events from a previous study and additional terms such as acute myocardial infarction, electrocardiogram (ECG) signs of myocardial infarction, and myocardial infarction were included (Table S3) [12].

The DRUG and REAC files were merged based on the same primary ID to create drug-adverse event pairs. Only drug-adverse event pairs in which the drug was suspected as the primary cause of the adverse event were analyzed. These drug-adverse event pairs are presented in a two-by-two contingency table (Table S4). From this table, the risk odds ratio (ROR) and 95% confidence intervals (CIs) were calculated. The ROR was calculated as (A/C)/(B/D), where A represents the number of cases of cardiotoxicity when PARP inhibitors were co-administered, B represents the number of cases of cardiotoxicity when PARP inhibitors were not co-administered, C represents the number of cases without cardiotoxicity when PARP inhibitors were co-administered, and D represents the number of cases without cardiotoxicity when PARP inhibitors were not co-administered. When calculating the ROR, if there were zero values in the numerator, 0.5 was added to all cells [13]. However, if both the numerator and denominator were equal to zero, the ROR was not computed. ROR values less than 1 were considered indicative of a decrease in the reporting of cardiotoxicity when PARP inhibitors were co-administered. Additional subgroup analyses were performed based on the type of anticancer agent (chemotherapy/bevacizumab, androgen receptor inhibitors, or immune checkpoint inhibitors) and patient sex and age (≥65 years or <65 years). The FAERS database analyses were conducted using SAS version 9.4 (SAS Institute Inc., Cary, NC, USA).

## 3. Results

### 3.1. Re-Analysis of the Previous Meta-Analysis

**Figure 1:**
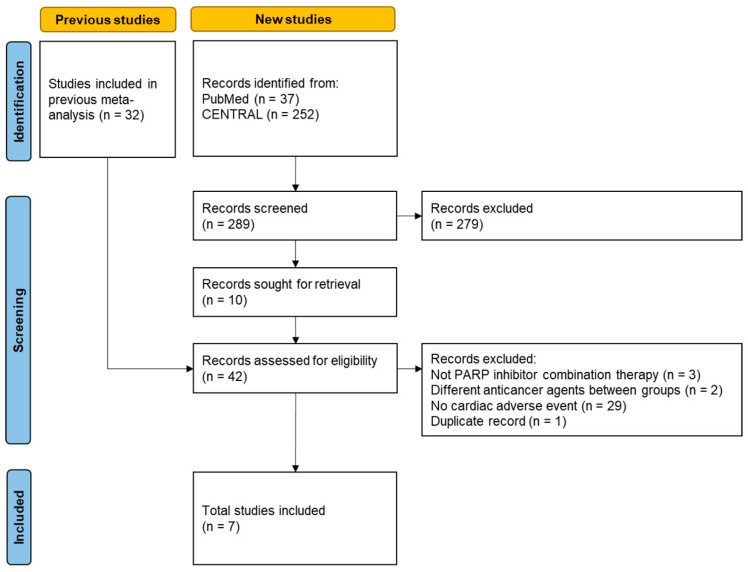
Flow diagram of study selection.

Figure 1 shows the flowchart of the current meta-analysis. Of the 289 newly searched records, 10 records were assessed for eligibility, but no additional studies were found. Ultimately, only seven studies from the previous meta-analysis were selected. The characteristics of the selected studies are summarized in Table 1 [14,15,16,17,18,19,20].

**Figure 2:**
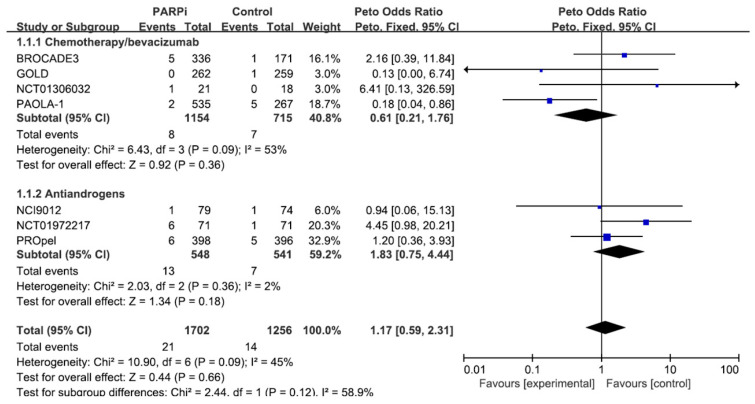
Effect of co-administration of PARP inhibitors with anti-cancer agents on cardiac adverse events.

Figure 2 presents a forest plot illustrating the impact of co-administration of PARP inhibitors. The co-administration of PARP inhibitors with anticancer agents did not show a statistically significant impact on cardiac adverse events (Peto OR = 1.17; *p* = 0.66). A subgroup analysis based on the type of anticancer agent revealed that co-administration of PARP inhibitors with chemotherapy/bevacizumab decreased the risk of cardiac adverse events (Peto OR = 0.61; *p* = 0.36), while co-administration of PARP inhibitors with antiandrogens increased the risk of cardiac adverse events (Peto OR = 1.83; *p* = 0.18); however, none of these findings reached statistical significance.

**Figure 3:**
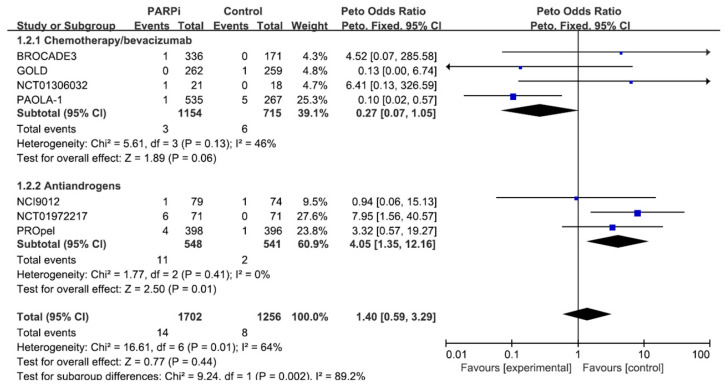
Effect of co-administration of PARP inhibitors with anticancer agents on grade ≥ 3 cardiac adverse events.

Figure 3 shows the results of the impact of PARP inhibitors on grade 3 or higher cardiotoxicity. Co-administration of PARP inhibitors with antiandrogens significantly increased the risk of cardiac adverse events (Peto OR = 4.05; *p* = 0.01). However, the effect of PARP inhibitors when used with chemotherapy/bevacizumab anticancer agents remained statistically non-significant (Peto OR = 0.27; *p* = 0.06).

### 3.2. Analysis of the FAERS Database

**Figure 4:**
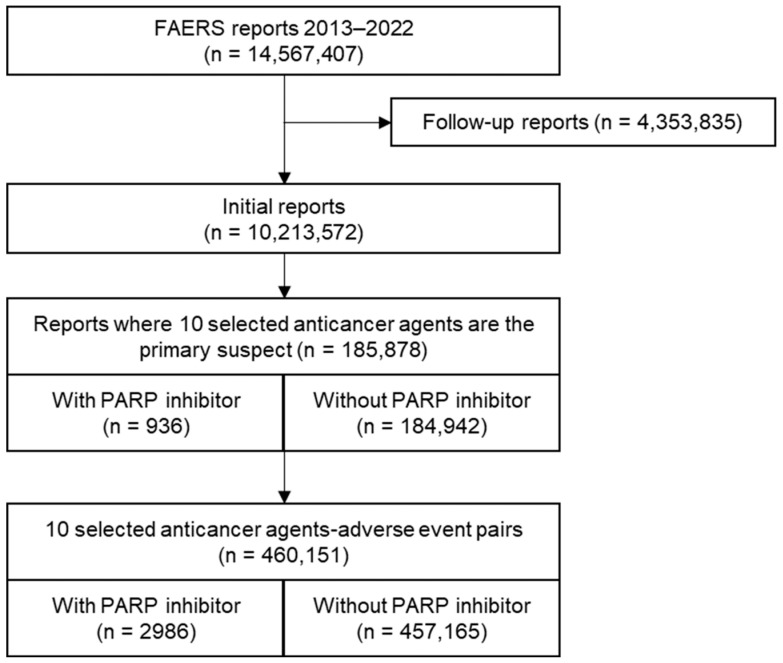
Flowchart for FAERS data processing.

Figure 4 shows the flowchart of data processing. The total number of adverse event reports was 14,567,407. After excluding follow-up reports, 185,878 reports in which 10 commonly co-administered anticancer agents were suspected of being the primary cause of the adverse events were selected. The list of adverse events was paired with 10 anticancer agents. A total of 2986 and 457,165 paired events were obtained with PARP inhibitors and without PARP inhibitors, respectively. The demographic characteristics based on the presence or absence of co-administration of PARP inhibitors with the 10 anticancer agents are presented in Table S5.

The ROR values are presented in Table 2. A total of 19 cases of cardiac adverse events were reported with co-administration of PARP inhibitors. The co-administration of PARP inhibitors with anticancer agents was associated with a decreased risk of cardiac adverse events (ROR = 0.585; 95% CI, 0.372–0.919).

For chemotherapy/bevacizumab, combination with PARP inhibitors significantly decreased the risk of cardiac adverse events (ROR = 0.352; 95% CI, 0.194–0.637). On the other hand, for co-administration of PARP inhibitors with antiandrogens, the ROR was 3.496 (95% CI, 1.539–7.942), suggesting a significant increase in the risk of cardiac adverse events when these drugs are co-administered with PARP inhibitors. The ROR for immune checkpoint inhibitors co-administered with PARP inhibitors was 0.606 (95% CI, 0.151–2.432), indicating a non-significant effect on cardiac adverse events. Detailed results for the individual drugs are provided in Table S6.

Table S7 shows the results of a subgroup analysis based on patient sex and age. In most subgroup analyses, the results were not statistically significant due to the small number of cases within each subgroup. The combination of PARP inhibitors with immune checkpoint inhibitors did not show a statistically significant association with cardiac adverse events in any subgroup analysis. However, the combination of PARP inhibitors with chemotherapy/bevacizumab significantly decreased the risk of cardiac adverse events in females and patients aged < 65 years (ROR, 0.335; 95% CI, 0.174–0.647 [female] and ROR, 0.348; 95% CI, 0.144–0.839 [age < 65 years]). On the other hand, the co-administration of PARP inhibitors with antiandrogens significantly increased the risk of cardiac adverse events in males and patients aged < 65 years (ROR, 3.733; 95% CI, 1.519–9.174 [male] and ROR, 8.008; 95% CI, 1.033–62.057 [age < 65 years]).

## 4. Discussion

This study investigated the potential cardioprotective effects of PARP inhibitors when used in combination with various anticancer agents through a re-analysis of a previously conducted meta-analysis and an analysis of the FAERS database over a 10-year period. The present meta-analysis and FAERS database analysis revealed that PARP inhibitors showed cardioprotective effects when combined with conventional anticancer agents. This result is in accordance with the findings of previous animal studies that have mentioned the cardioprotective effects of PARP inhibitors [5,7,8]. The mechanism behind these protective effects could be attributed to the ability of PARP inhibitors to limit or inhibit decreases in myocardial NAD+ and ATP and modulating cellular stress responses, potentially reducing oxidative stress and protecting cardiomyocytes [3,21].

In contrast to their cardioprotective role when combined with chemotherapy/bevacizumab, PARP inhibitors seemed to increase the risk of cardiac adverse events when used in conjunction with antiandrogens. Abiraterone irreversibly inhibits the cytochrome P450 17 alpha-hydroxylase (CYP17) enzyme in the adrenal glands, selectively suppressing androgen synthesis [22]. This inhibition of CYP17 leads to decreased cortisol levels and increased production of mineralocorticoids due to the rise in adrenocorticotropic hormone [23]. This phenomenon, which results in hypermineralocorticism induced by abiraterone, appears to be associated with the risk of atrial fibrillation and congestive heart failure when abiraterone is used alone [23,24]. However, the mechanism behind the increased risk of cardiac adverse events with the concomitant use of PARP inhibitors is not yet understood. Perhaps an important role of PARP in DNA repair of PARP at its normal levels that does not deplete NAD+ or ATP to a significant extent and facilitates myocyte and endothelial cell survival is hindered. This inhibition, coupled with the potential cardiac toxicity of abiraterone, could result in a synergistic effect. This finding suggests that further investigation into the intricate interactions of PARP inhibitors, antiandrogens, and cardiac tissue is warranted.

There is scarce information regarding the cardiotoxicity associated with the combination of immune checkpoint inhibitors and PARP inhibitors. In general, the frequency of cardiotoxicity due to immune checkpoint inhibitors is not particularly high (approximately 1%) [25]. The most common form is myocarditis involving CD4+-mediated T-cell inflammation rather than the cardiac adverse events defined in our study. Immune checkpoint inhibitors activate the NLRP3 and MyD88 pathways associated with inflammatory responses in myocardial tissue [26]. Specifically, NLRP3 induces cytosolic damage and hypertrophy through the expression of inflammatory cytokines [26]. Heart failure and myocardial infarction are rare events with incidences of approximately 0.3% and 0.4%, respectively [27]. Given these low occurrence rates and the fact that the anticancer mechanism of immune checkpoint inhibitors is linked to proinflammation rather than direct cytotoxicity, it is unlikely that the impact of PARP inhibitors would be significant. Resveratrol, a natural compound, has been suggested for consideration in combination therapy as it can enhance the response of cancer cells to PARP inhibitors and reduce inflammation in the heart mediated by NLRP3 [28,29].

Considering the potential impact of age and gender differences on the results, a subgroup analysis was performed. In previous animal studies, inhibition of PARP showed protective effects only in male animals, while there was no benefit observed in female animals [30,31]. Female sex itself offered inherent protection that was not augmented by inhibiting PARP activity [32]. Mabley et al. have suggested the cooperative interaction of PARP and the estrogen receptor (ER) α with DNA as a plausible explanation for this phenomenon [31]. In an in vitro study, the presence of an estrogen ligand induced conformational changes in ERα, leading to the formation of a more stable ERα-PARP-DNA complex. This increased stability could potentially impede PARP from accessing and recognizing DNA breakpoints [31]. Therefore, deriving additional benefits from the use of PARP inhibitors might be challenging for female animals. However, unlike animal studies, our research demonstrated a protective effect of PARP inhibitors in females as well. It is likely that the interplay of various intricate factors, including biological dissimilarities and complexities in drug metabolism and effects, contributes to these variations [33]. Furthermore, our data were obtained from patients with a relatively advanced age. Menopause generally begins between the ages of 45 and 55 years, thereby increasing the possibility of diminished effects of estrogen influence. Furthermore, the cellular poly(ADP-ribosyl)ation capacity decreases with age [3], suggesting that the impact of PARP inhibitors could diminish with increasing age. In accordance with this, our study did not observe significant cardiac protective benefits from PARP inhibitor treatment among participants aged 65 years and older. However, the findings of this subgroup analysis may not have shown statistical significance due to reduced event counts, emphasizing the need for cautious interpretation.

The results of this re-analysis of a previous meta-analysis and FAERS database analysis must be interpreted with caution owing to the limitations of this work. First, the results of a meta-analysis can be influenced by the studies included, and studies with unreported cardiac adverse events that were not considered adverse events of interest might have been excluded from the analysis. Second, the FAERS data are susceptible to reporting bias, which potentially could have resulted in omissions or errors in the data. This becomes problematic in cases involving the co-administration of PARP inhibitors, where instances may have arisen in which the concurrent use of PARP inhibitors as co-administered drugs was not explicitly reported. Third, a subgroup analysis based on age and sex was conducted; however, a significant amount of information on other factors that could impact the occurrence of cardiac adverse events was missing, such as patients’ underlying cardiac conditions and the duration of anticancer therapy. As a result, these factors were not incorporated into the analysis. Claims data accumulated from the government’s health insurance system should be used to conduct a sophisticated analysis considering multiple factors. Finally, due to discrepancies between the actual occurrence of adverse events and their reporting, it is not possible to calculate a reduction in the incidence rate of cardiac adverse events.

## 5. Conclusions

Our study indicates that PARP inhibitors show cardioprotective effects when used with conventional anticancer agents. However, the interaction between antiandrogens and PARP inhibitors increases the risk of cardiac adverse events, necessitating caution. These findings contribute to a more comprehensive understanding of the cardioprotective role of PARP inhibitors when co-administered with other anticancer agents, offering valuable insights for future therapeutic approaches. However, further research is warranted to elucidate the intricate mechanisms underlying these observed effects and to guide the clinical application of PARP inhibitors in the realm of cardioprotection.

## Figures and Tables

**Table 1:** Studies included in the meta-analysis.

Study Name (First Author, Year)	Cancer Type	With a PARP Inhibitor	Without a PARP Inhibitor	Number of Patients
NCT01306032 (Kummar et al., 2016) [14]	Breast	Cyclophosphamide + Veliparib (60 mg once daily)	Cyclophosphamide	39
GOLD (Bang et al., 2017) [15]	Gastric	Paclitaxel + Olaparib (100 mg twice daily)	Paclitaxel + Placebo	521
NCI9012 (Hussain et al., 2018) [16]	Prostate	Abiraterone + Veliparib (300 mg twice daily)	Abiraterone	153
NCT01972217 (Clarke et al., 2018) [17]	Prostate	Abiraterone + Olaparib (300 mg twice daily)	Abiraterone + Placebo	142
PAOLA-1 (Ray-Coquard et al., 2019) [18]	Ovarian	Bevacizumab + Olaparib (300 mg twice daily)	Bevacizumab + Placebo	802
BROCADE3 (Diéras et al., 2020) [19]	Breast	Carboplatin + Paclitaxel + Veliparib (120 mg twice daily)	Carboplatin + Paclitaxel + Placebo	507
PROpel (Thiery-Vuillemin et al., 2022) [20]	Prostate	Abiraterone + Olaparib (300 mg twice daily)	Abiraterone + Placebo	794

PARP, Poly(adenosine diphosphate [ADP] ribose) polymerase.

**Table 2:** ROR for cardiac adverse events with co-administration of PARP inhibitors and anticancer agents.

	Number of Cardiac Adverse Events with PARP Inhibitors	ROR (95% CI)
Total	19	0.585 (0.372–0.919)
Chemotherapy/bevacizumab	11	0.352 (0.194–0.637)
Immune checkpoint inhibitors	2	0.606 (0.151–2.432)
Antiandrogens	6	3.496 (1.539–7.942)

CI, confidence interval; PARP, Poly(adenosine diphosphate [ADP] ribose) polymerase; ROR, reporting odds ratio.

## Data Availability

Dataset available on request from the authors.

## Supplementary Materials

The following supporting information can be downloaded at: https://www.mdpi.com/article/10.3390/jcm13051218/s1, Table S1: Population, intervention, comparison, outcome, and study design of the meta-analysis; Table S2: Search strategy; Table S3: Preferred terms associated with cardiotoxicity; Table S4: Two-by-two contingency table; Table S5: Demographic characteristics for the 10 anticancer agents; Table S6: Reporting odds ratio (ROR) for cardiac adverse events with co-administration of PARP inhibitor and anticancer agents (individual drugs); Table S7: Reporting odds ratio (ROR) for cardiac adverse events with co-administration of PARP inhibitor and anticancer agents (Subgroup analysis).
