# Supplementary material for: Cardioprotective Effects of PARP Inhibitors: A Re-Analysis of a Meta-Analysis and a Real-Word Data Analysis Using the FAERS Database
Source: J Clin Med. 2024 Feb 21;13(5):1218. doi: 10.3390/jcm13051218 (PMC10932277; doi:10.3390/jcm13051218)
Supplement: Supplementary file 1 [file jcm-13-01218-s001.zip › jcm-2798457-supplementary.pdf]

Table S1. Population, intervention, comparison, outcome, and study design of the meta-analysis

PICOS	Definition	Inclusion criteria
Population	Patients with cancer	Any type of cancer
Intervention	Anticancer agents + PARP inhibitors	Use of one PARP inhibitor (niraparib, olaparib, rucaparib, talazoparib, veliparib)
Comparison	Anticancer agents ± placebo	Same anticancer agents as the anticancer agents administered in the intervention group
Outcome	Adverse cardiac events	MedDRA preferred terms related to adverse cardiac events (Table S2)
Study design	Randomized controlled trial	Randomized controlled trial

PARP, Poly(adenosine diphosphate [ADP] ribose) polymerase

Table S2. Search strategy

Database	Search terms	Filter
PubMed	("Poly(ADP-ribose) Polymerase Inhibitors"[Mesh] OR PARPi OR 'PARP inhibitor' OR olaparib OR niraparib OR rucaparib OR talazoparib OR veliparib)	Publication date: 1st May 2022 to 2nd June 2023<br>Article type: randomized controlled trial
Cochrane Central Register of Controlled Trials	Same as above	Date: 1st May 2022 to 2nd June 2023

Table S3. Preferred terms associated with cardiotoxicity

MedDRA preferred terms	
Acute left ventricular failure	Cardiomyopathy
Cardiac failure	Cardiomyopathy acute
Cardiac failure acute	Cardiotoxicity
Cardiac failure chronic	Congestive cardiomyopathy
Cardiac failure congestive	Cytotoxic cardiomyopathy
Chronic left ventricular failure	Ejection fraction abnormal
Ejection fraction decreased	Eosinophilic myocarditis
Left ventricular failure	Non-obstructive cardiomyopathy
Low cardiac output syndrome	Echocardiogram abnormal
Ventricular failure	Multiple gated acquisition scan abnormal
Cardiac index decreased	Nuclear magnetic resonance imaging thoracic abnormal
Cardiac output decreased	Scan myocardial perfusion abnormal
Cardiac ventriculogram abnormal	Ventricular hypokinesia
Cardiac ventriculogram left abnormal	Acute myocardial infarction
Cardiomegaly	ECG signs of myocardial infarction
Left ventricular dysfunction	Myocardial infarction
Systolic dysfunction	
Ventricular dysfunction	

ECG, electrocardiogram

Table S4. Two-by-two contingency table

	With PARP inhibitors	Without PARP inhibitors
With cardiotoxicity	A	B
Without cardiotoxicity	C	D

PARP, Poly(adenosine diphosphate [ADP] ribose) polymerase

Table S5. Demographic characteristics for the 10 anticancer agents

Abiraterone acetate	Without PARP inhibitor	With PARP inhibitor	Atezolizumab	Without PARP inhibitor	With PARP inhibitor
N	22418	49	N	8665	39
Age†	72.4±16.1	71.6±8.6	Age†	54.5±25.8	59.8±10.9
Sex			Sex		
Male	20649 (92.1)	46 (93.9)	Male	4260 (49.2)	8 (20.5)
Female	128 (0.6)	0	Female	2845 (32.8)	29 (74.4)
Missing	1641 (7.3)	3 (6.1)	Missing	1560 (18.0)	2 (5.1)
Bevacizumab	Without PARP inhibitor	With PARP inhibitor	Carboplatin	Without PARP inhibitor	With PARP inhibitor
N	25825	281	N	23228	240
Age†	58.8±17.4	59.5±13.3	Age†	57.1±20.5	63.0±15.3
Sex			Sex		
Male	7400 (28.7)	2 (0.7)	Male	7255 (31.2)	13 (5.4)
Female	10454 (40.5)	258 (91.8)	Female	10041 (43.2)	199 (82.9)
Missing	7971 (30.9)	21 (7.5)	Missing	5932 (25.5)	28 (11.7)
Doxorubicin	Without PARP inhibitor	With PARP inhibitor	Durvalumab	Without PARP inhibitor	With PARP inhibitor
N	16349	61	N	4442	26
Age†	49.0±22.4	55.8±12.1	Age†	64.3±15.0	59.0±19.2
Sex			Sex		
Male	4884 (29.9)	0	Male	2449 (55.1)	8 (30.8)
Female	6437 (39.4)	57 (93.4)	Female	1249 (28.1)	17 (65.4)
Missing	5028 (30.8)	4 (6.6)	Missing	744 (16.7)	1 (3.8)
Nivolumab	Without PARP inhibitor	With PARP inhibitor	Paclitaxel	Without PARP inhibitor	With PARP inhibitor
N	35979	84	N	17953	89
Age†	61.3±17.1	62.2±11.8	Age†	59.9±13.9	61.2±11.7
Sex			Sex		
Male	19376 (53.9)	25 (29.8)	Male	4240 (23.6)	10 (11.2)
Female	10924 (30.4)	59 (70.2)	Female	10730 (59.8)	74 (83.1)
Missing	5679 (15.8)	0	Missing	2983 (16.6)	5 (5.6)
Pembrolizumab	Without PARP inhibitor	With PARP inhibitor	Temozolomide	Without PARP inhibitor	With PARP inhibitor
N	21133	33	N	8950	34
Age†	61.1±20.8	61.9±12.5	Age†	52.9±19.1	44.0±12.7
Sex			Sex		
Male	10831 (51.3)	8 (24.2)	Male	4138 (46.2)	18 (52.9)
Female	8668 (41.0)	24 (72.7)	Female	3485 (38.9)	7 (20.6)
Missing	1634 (7.7)	1 (3.0)	Missing	1327 (14.8)	9 (26.5)

PARP, Poly(adenosine diphosphate [ADP] ribose) polymerase

†The analysis was performed by excluding missing values.

Table S6. Reporting odds ratio (ROR) for cardiac adverse events with co-administration of PARP inhibitor and anticancer agents (individual drugs)

Class	Anticancer agent	Number of cardiac adverse events with PARP inhibitor	ROR (95% CI)
Chemotherapy/ bevacizumab	Bevacizumab	8	0.803 (0.398–1.617)
	Carboplatin	2	0.504 (0.125–2.026)
	Paclitaxel	0	0.322 (0.020–5.170)
	Doxorubicin	1	0.178 (0.025–1.273)
	Temozolomide	0	2.070 (0.127–33.879)
Antiandrogens	Abiraterone	6	3.496 (1.539–7.942)*
Immune checkpoint inhibitors	Nivolumab	2	1.452 (0.360–5.863)
	Atezolizumab	0	0.642 (0.040–10.396)
	Durvalumab	0	1.329 (0.081–21.782)
	Pembrolizumab	0	0.581 (0.036–9.357)

CI, confidence interval; PARP, Poly(adenosine diphosphate [ADP] ribose) polymerase; ROR, reporting odds ratio

*Statistically significant ($p < 0.05$)

Table S7. Reporting odds ratio (ROR) for cardiac adverse events with co-administration of PARP inhibitor and anticancer agents (Subgroup analysis)

	**Number of cardiac adverse events with PARP inhibitor**	**ROR (95%CI)**
**Male**		
Total	9	2.638 (1.359–5.121)*
Chemotherapy/bevacizumab	2	1.400 (0.346–5.673)
Antiandrogens	5	3.733 (1.519–9.174)*
Immune checkpoint inhibitors	2	2.699 (0.664–10.972)
**Female**		
Total	9	0.351 (0.182–0.677)*
Chemotherapy/bevacizumab	9	0.335 (0.174–0.647)*
Antiandrogens	0	NA
Immune checkpoint inhibitors	0	0.248 (0.015–3.986)
**Age ≥ 65 years**		
Total	5	0.483 (0.200–1.166)
Chemotherapy/bevacizumab	2	0.252 (0.063–1.011)
Antiandrogens	3	2.720 (0.855–8.661)
Immune checkpoint inhibitors	0	0.300 (0.019–4.815)
**Age < 65 years**		
Total	8	0.576 (0.287–1.156)
Chemotherapy/bevacizumab	5	0.348 (0.144–0.839)*
Antiandrogens	1	8.008 (1.033–62.057)*
Immune checkpoint inhibitors	2	1.824 (0.450–7.384)

CI, confidence interval; PARP, Poly(adenosine diphosphate [ADP] ribose) polymerase; ROR, reporting odds ratio

*Statistically significant ($p < 0.05$)
